# Cytotoxicity of Cultured Canine Primary Hepatocytes Exposed to Itraconazole Is Decreased by Pre-treatment With Glutathione

**DOI:** 10.3389/fvets.2021.621732

**Published:** 2021-02-18

**Authors:** Natalie M. Kirk, Miranda D. Vieson, Kim A. Selting, Jennifer M. Reinhart

**Affiliations:** ^1^Department of Veterinary Clinical Medicine, College of Veterinary Medicine, University of Illinois, Urbana, IL, United States; ^2^Department of Pathobiology, College of Veterinary Medicine, University of Illinois, Urbana, IL, United States

**Keywords:** hepatocytes, hepatotoxicity, glutathione, itraconazole, canine

## Abstract

**Objective:** To identify the effect of glutathione (GSH) on cell survival in a novel *in vitro* model of itraconazole (ITZ)-associated hepatotoxicity using canine primary hepatocytes.

**Sample:** Commercially sourced, cryopreserved male dog (Beagle) primary hepatocytes from a single donor.

**Procedures:** Using a sandwich culture technique, canine primary hepatocytes were exposed to serial dilutions of ITZ. Calcein AM, 3-(4,5-dimethylthiazol-2-yl)-2, 5-diphenyltetrazolium bromide (MTT), and neutral red were investigated as potential cell viability assays. Hepatocytes were then pre-incubated with GSH, exposed to serial dilutions of ITZ, and cell viability determined at 4 and 24 h post-ITZ exposure. Each condition was performed in technical triplicate and the effect of time, GSH concentration, and ITZ concentration on % cytotoxicity assessed using a multivariate linear regression model. Tukey's *post-hoc* test was used to detect individual differences.

**Results:** The neutral red cell cytotoxicity assay was chosen based on its superior ability to detect dose-dependent changes in viability. Hepatocyte cytotoxicity significantly increased with ITZ concentration (*P* < 0.001) and time (*P* = 0.004) and significantly decreased with GSH treatment (*P* < 0.001).

**Conclusions and Clinical Relevance:** This *in vitro* model demonstrates dose- and time-dependent ITZ-induced cytotoxicity, which is similar to clinical changes observed in canine patients and in *in vivo* rodent studies. Pre-treating with GSH is protective against *in vitro* cell death. These results suggest that GSH precursors may have a role in the management or prevention of ITZ-associated hepatotoxicity in dogs. Clinical trials are needed to evaluate their utility for this adverse drug reaction.

## Introduction

Itraconazole (ITZ) is an azole antifungal drug used to treat a variety of fungal infections in dogs, including dermatophytosis, malasseziosis, systemic mycoses such as blastomycosis and histoplasmosis, and mycoses caused by opportunistic fungi (1). The azoles' antifungal activity is attributable to inhibition of ergosterol by targeting the cytochrome P450-dependent sterol 14α-demethylase (2). The most commonly used azoles in veterinary medicine are ITZ, fluconazole, and ketoconazole. Itraconazole is more selective than ketoconazole (KTZ), leading to fewer adverse effects, and has a broader spectrum of action than both KTZ and fluconazole (3, 4). Additionally, ITZ is more efficacious than ketoconazole in treating a variety of fungal infections in animal models (5, 6).

In veterinary medicine, ITZ is effective in treating systemic infections with *Blastomyces dermatitidis, Histoplasma capsulatum, Cryptococcus neoformans*, and *Coccidioides immitis*, among others (1, 7–9). These soil-borne, dimorphic fungi have regional distributions in the United States and cause serious, sometimes fatal disease in animals. People can also be infected with these fungi with outbreaks occasionally occurring concurrently in human and canine populations (10, 11). In a study of human and canine blastomycosis cases in Illinois, trends in incidence and distribution were similar for humans and dogs, indicating that dogs may be a good sentinel species for human infections (12). Because these systemic mycoses cause significant morbidity and mortality, a safe and effective treatment is essential.

Despite its efficacy, ITZ is associated with a number of adverse reactions in dogs including nausea, vomiting, lethargy, anorexia, ulcerative dermatitis, and hepatotoxicity (7, 8). Anorexia is most common and is often associated with increases in alanine aminotransferase (ALT). Alanine aminotransferase increases are reported in 12–42% of dogs depending on the dose and duration of treatment (7–9). In these studies, the definition of hepatotoxicity varied from any increase in ALT to extreme increases associated with the above clinical signs. Oftentimes, ALT increase is asymptomatic; however, based on clinical experience, hepatotoxicity leading to abnormal hepatic function does occur. The prevalence of liver dysfunction associated with ITZ therapy in dogs is not reported. In a systemic review and meta-analysis of humans experiencing similar adverse reactions to ITZ, the pooled risk of developing elevated serum liver enzymes not requiring a treatment change was 17.4%, while development of abnormal liver function tests necessitating discontinuation of treatment was 1.5% (13). Although drug discontinuation generally results in reversal of these changes in dogs, switching antifungal drugs can negatively affect the course of disease if alternative drugs are less effective or cause adverse reactions. Due to its efficacy, continuing ITZ treatment while managing hepatotoxicity may be preferred in many cases.

Mechanisms of ITZ-associated hepatotoxicity in people and animals are poorly understood. However, data from *in vitro* and *in vivo* rodent studies implicate oxidative stress as a likely contributor to its pathogenesis (14, 15). Of particular interest, hepatic glutathione (GSH), an essential, endogenous antioxidant, is depleted during ITZ administration in rats (15). Thus, GSH depletion may have an important role in the pathogenesis of ITZ-associated hepatotoxicity and is also a prime target for therapy.

Two GSH precursors, SAMe and N-acetylcysteine, can be administered easily to dogs and are frequently used in veterinary medicine. Both are documented to increase systemic GSH concentration in this species (16, 17). In people, SAMe has been effective in treating alcohol-induced (18) and drug-induced liver injury (19). SAMe combined with another antioxidant, silybin, is effective in preventing lomustine-induced hepatotoxicity in canine cancer patients (20). This combination has also been shown to increase GSH and decrease the production of pro-inflammatory mediators in canine hepatocytes exposed to acetaminophen (21, 22). N-acetylcysteine is the treatment of choice for acetaminophen toxicosis (23) and is often used in combination with other hepatoprotectants in dogs with liver failure of various etiologies (24).

These successes have led to clinical recommendations for the use of GSH precursors in the management of ITZ-associated hepatotoxicity in dogs, despite the lack of evidence to support efficacy in either preventing or treating this adverse drug reaction. These products are expensive and can cause inappetence, so scientific evidence is needed to provide a basis for the use of GSH precursors in canine patients with ITZ-associated hepatotoxicity. The first aim of this study was to develop an *in vitro* model to assess the toxic effects of ITZ in cryopreserved canine hepatocytes. The second aim was to evaluate the effect of GSH on this toxicity. We hypothesized that (1) ITZ would induce measurable cytotoxicity in primary canine hepatocytes and (2) GSH treatment would decrease that toxicity.

## Materials and Methods

### Cell Culture

The following methods were modified from protocols provided by the supplier. Materials were obtained from ThermoFisher Scientific (Waltham, MA) except where noted. Commercially sourced, cryopreserved male dog (Beagle) primary hepatocytes from a single donor were thawed in a 37°C water bath for <2 min and resuspended in 50 mL of thawing/plating medium (William's E medium with 5% fetal bovine serum, 1 μM DMSO, 1% penicillin/streptomycin, 4 μg/mL human recombinant insulin, 2 mM GlutaMAX, and 15 mM HEPES at pH 7.4). Following resuspension, the cells were centrifuged at 60 x *g* for 4 min and counted using a hemocytometer and the Trypan Blue dye exclusion method. The cells were then plated in a sandwich configuration onto pre-collagen I coated, sterile culture plates and incubated at 37°C for 4 h. After incubation, the supernatant was discarded and cold ECM gel from Engelbreth-Holm-Swarm murine sarcoma diluted to 0.35 mg/mL in incubation medium (William's E medium with 0.1 μM dexamethasone in DMSO, 0.5% penicillin/streptomycin, 6.25 μg/L human recombinant insulin, 6.25 μg/mL human transferrin, 6.25 ng/mL selenous acid, 1.25 mg/mL bovine serum albumin, 5.35 μg/mL linoleic acid, 2 mM GlutaMAX, and 15 mM HEPES at pH 7.4) was applied over the cells prior to an overnight incubation at 37°C.

### Maintenance of Cell Phenotype

To determine maintenance of biochemical phenotype, cryopreserved primary canine hepatocytes plated as described above were incubated for 24, 48, or 72 h. No-cell wells were included as controls. Albumin production and maintenance of morphologic features were assessed as these are common markers used to evaluate hepatic phenotype *in vitro* (25). At each time point, supernatants were collected and frozen at −80°C. Frozen supernatants were later thawed on ice and albumin concentrations determined using a canine-specific albumin enzyme linked immunosorbent assay kit according to manufacturer's instructions (Eagle Biosciences, Amherst, NH). Absorbance was quantified using a SpectraMax iD3 multi-mode microplate reader and SoftMax Pro 7 Software (Molecular Devices, San Jose, CA) and concentrations calculated from a standard curve. To determine maintenance of cellular morphology, cells were examined for polygonality and presence of cell-to-cell junctions at 24, 48, and 72 h of incubation using the Invitrogen EVOS XL Core Cell Imaging System (ThermoFisher Scientific, Waltham, MA).

### Cell Viability Assay Optimization

Three viability assays were trialed during model development: calcein AM, 3-(4,5-dimethylthiazol-2-yl)-2, 5-diphenyltetrazolium bromide (MTT), and neutral red. For these tests, cells were cultured as described above and exposed to 0, 2, 10, or 50 μM ITZ diluted in DMSO for 4 h. DMSO concentration was below 1% for all treatments. Cells were plated at 5.0 × 10^5^ cells/ml for the calcein AM assay. For the MTT and neutral red assays, cells were plated at both 0.8 × 10^5^ cells/ml 2.4 × 10^5^ cells/ml to establish seeding density for subsequent experiments. A SpectraMax iD3 multi-mode microplate reader and SoftMax Pro 7 software (Molecular Devices, San Jose, CA) were used to measure absorbance (O.D.) or fluorescence (RFUs) depending on the assay. Each condition was performed in technical triplicate and % cytotoxicity calculated as:

$$\begin{array}{l} \%\;cytotoxicity=\\ \left(1-\;\frac{mean\;value_{treatment}-\;mean\;value_{blank}}{mean\;value_{untreated\;cells}-\;mean\;value_{blank}}\right)\;\times \;100\% \end{array}$$

The first assay tested was calcein AM, which is a non-fluorescent marker that permeates live, intact cells. Cytosolic esterases hydrolyze calcein AM into the fluorescent molecule calcein (26). Following incubation with ITZ, the cells were washed with incubation medium and incubated with diluted calcein AM (ThermoFisher Scientific; Waltham, MA) at 37° C for 30 min. Then, the cells were washed again, and fluorescence measured at an excitation wavelength of 485 nm and emission wavelength of 525 nm.

The second assay tested was MTT (ThermoFisher Scientific; Waltham, MA). MTT is reduced by a metabolically active cell into insoluble formazan via mitochondrial NADPH oxidoreductase (27). Following incubation with ITZ, the cells were washed and incubated with diluted MTT at 37° C for 4 h. Formazan was then solubilized by the addition of 0.1% DMSO and absorbance measured at 590 nm.

Lastly, neutral red uptake into lysosomes (28) was measured using a neutral red cell cytotoxicity assay kit (BioVision; Milpitas, CA) according to the manufacturer's instructions. Briefly, the cells were washed following the 4 h incubation with ITZ and then incubated for 2 h at 37°C with neutral red solution. The cells were washed again before adding the provided solubilization solution. Viable cells release neutral red into the supernatant after solubilization, which was then measured at an absorbance of 540 nm.

### Exposure of Cells to GSH and ITZ

Following plating of cells at a density of 2.4 × 10^5^ cells/ml and overnight incubation with the ECM gel overlay as described above, supernatants were removed. Cells were then treated with 100 μl of 0, 50, or 500 μM GSH (Sigma-Aldrich, St. Louis, MO) diluted in incubation medium, and incubated for 24 h. These concentrations were chosen based on GSH concentrations previously described for canine liver (29). After incubation, supernatants were removed, and cells were washed with 200 μl of incubation medium. Then cells were treated with 100 μl of 0, 2, 10, or 50 μM ITZ (Sigma-Aldrich, St. Louis, MO) diluted in incubation medium and DMSO (final concentration 1%) and incubated for 4 or 24 h. In dogs, peak plasma concentrations of ITZ are approximately 2 μM following oral administration (30). Intrahepatic accumulation is expected based on drug distribution studies (31), so concentrations of up to 50 μM were investigated. No-cell controls were included as blanks for viability assays. Cells treated for 1 h with 2% Triton-X were used as a positive control for cell death while untreated cells were used as a negative control. Cells exposed to DMSO only were used as a vehicle control and included in the analysis as the 0 μM ITZ/0 μM GSH condition.

The neutral red cell cytotoxicity assay was used to determine cell viability at 4- and 24-h post ITZ exposure. Following neutral red solubilization, the absorbance was quantified, and cytotoxicity calculated as described previously. Each condition was performed in technical triplicate.

### Statistical Analysis

Selection of the most appropriate viability assay was based on demonstration of dose-dependent cytotoxicity in cells exposed to ITZ, as this effect is expected based on clinical evidence in dogs and experimental rodent studies (32, 33). For maintenance of phenotype testing, an unpaired *t*-test was used to compare albumin production by hepatocytes and media control wells. Changes in cellular morphology were assessed qualitatively. For the cytotoxicity results, the effect of time, GSH concentration, and ITZ concentration on % cytotoxicity was assessed using a multivariate linear regression model. Tukey's *post-hoc* test was used to detect individual differences in % cytotoxicity between ITZ concentrations for both the 4 and 24 h data. Results are reported as the mean ± standard deviation. All analyses were performed using Prism 8 (GraphPad Software, San Diego, CA) and JMP 14 (SAS Institute, Cary, NC). Statistical significance was set at *P* < 0.05.

## Results

### Maintenance of Hepatic Phenotype

**Figure 1:**
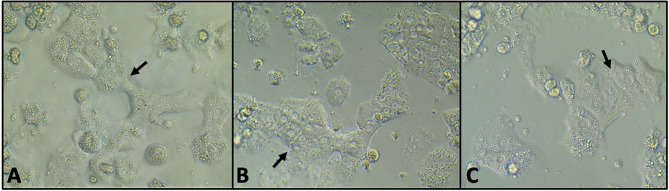
Phase contrast microscopy of untreated hepatocytes at 24 h **(A)**, 48 h **(B)**, and 72 h **(C)** of incubation. The cells maintained cell-to-cell adhesion (arrows) and the polygonal morphology characteristic of hepatocytes.

Albumin was detectable at all time points in significantly greater amounts than media controls (*P* < 0.001). Hepatocytes maintained cell-to-cell adhesion and their expected polygonal morphology at all time points (Figure 1).

### Cell Viability Assay Optimization

**Figure 2:**
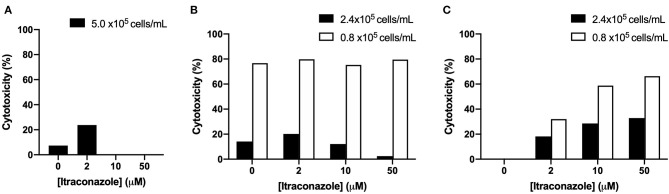
Percent cytotoxicity of hepatocytes treated with ITZ for 4 h and viability detected using **(A)** calcein AM, **(B)** MTT, or **(C)** neutral red.

The results of cell viability optimization are presented graphically in Figure 2. A dose-dependent increase in hepatocyte cytotoxicity was not detected after 4 h of ITZ exposure using calcein AM or MTT assays. In contrast, dose-dependent cytotoxicity was detected after 4 h of ITZ exposure using the neutral red assay. For both the MTT and neutral red assays, overall viability was lower in cells plated at 0.8 × 10^5^ cells/ml compared to 2.4 × 10^5^ cells/ml.

### The Effect of ITZ and GSH on Cryopreserved Canine Hepatocytes

**Figure 3:**
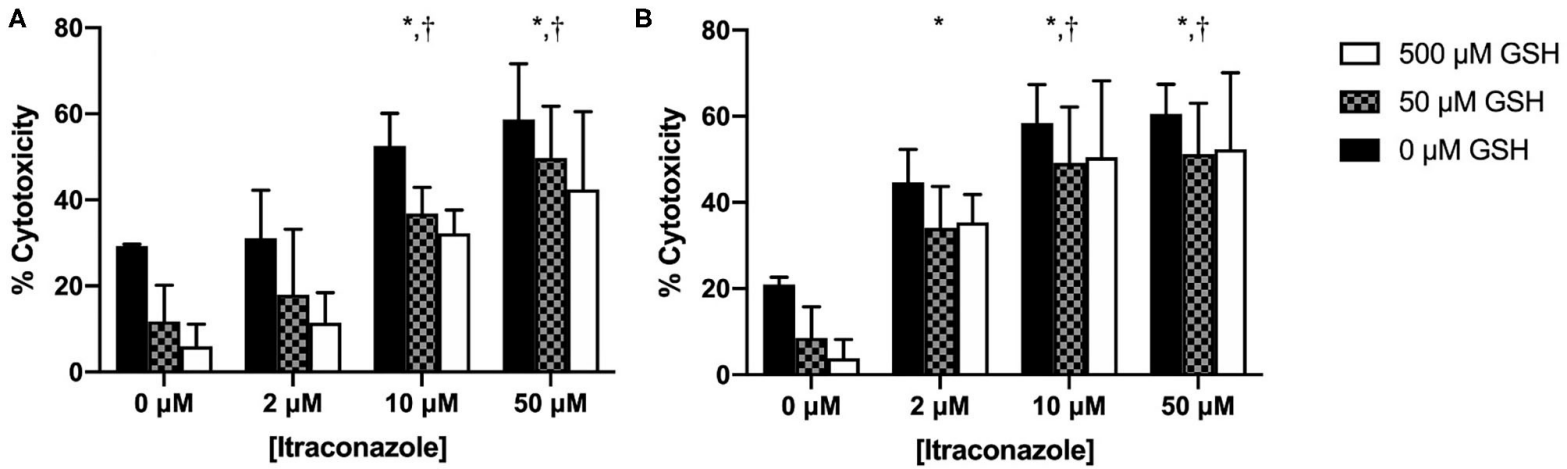
Percent cytotoxicity of hepatocytes treated with GSH and ITZ **(A)** 4 h and **(B)** 24 h after ITZ exposure. *, significantly different from 0 μM ITZ; ^†^, significantly different from 2 μM ITZ.

Cytotoxicity results are graphically presented in Figure 3. Hepatocyte cytotoxicity significantly increased with time (*P* = 0.004) and ITZ concentration (*P* < 0.001). Cytotoxicity significantly decreased with increasing GSH concentration (*P* < 0.001). A significant interaction was identified between time and ITZ concentration (*P* = 0.014). No other significant interactions between investigated variables were present (*P* = 0.306 – 0.999).

At 4 h, % cytotoxicity was greater for cells treated with 10 and 50 μM ITZ vs. control (*P* < 0.001) and for cells treated with 10 μM (*P* = 0.002) and 50 μM (*P* < 0.001) vs. 2 μM ITZ. At 24 h, % cytotoxicity was greater for cells treated with 2, 10, and 50 μM ITZ vs. control (*P* < 0.001). Cytotoxicity was also greater for cells treated with 10 μM (*P* = 0.032) and 50 μM (*P* = 0.013) vs. 2 μM. Thus, the effect of ITZ on % cytotoxicity appeared to be dose-dependent at both time points. No other significant differences were found for individual comparisons between ITZ concentrations at either time point.

## Discussion

In this study, we showed that cryopreserved canine primary hepatocytes in a sandwich culture are an appropriate *in vitro* model for canine ITZ-associated hepatotoxicity. Over the course of 72 h, cells maintained lineage-specific albumin production and morphologic features, confirming maintenance of hepatocyte phenotype over the course of the experiment. Detection of cell death was best accomplished by measuring the uptake of neutral red by viable cells. Using this detection method, ITZ caused significant dose- and time-dependent cytotoxicity *in vitro* in primary canine hepatocytes, which mimics the dose-dependent hepatotoxicity seen *in vivo* (7). Comparable results have been demonstrated in rodent models (32, 33). Pre-treatment of the cells with GSH for 24 h prior to ITZ exposure had a significant protective effect against ITZ-associated cytotoxicity. These results suggest that GSH or its precursors may play a role in preventing or treating this toxicity *in vivo*.

During the model development stage, we investigated three different markers of cell viability: calcein AM, MTT, and neutral red. Of the three viability assays, only neutral red yielded reproducible results depicting the expected dose-dependent increase in hepatocyte toxicity after 4 hours of ITZ exposure. For the neutral red assay, a seeding density of 2.4 × 10^5^ cells/ml was selected because viability was markedly decreased at 0.8 × 10^5^ cells/ml, likely as a result of low signal detection. Based on these findings, neutral red uptake with a seeding density of 2.4 × 10^5^ cells/ml appears to be the most suitable measurement of cell viability in canine hepatocytes exposed to ITZ. This protocol was used in our subsequent investigation of GSH and can be used in future experiments.

We also documented a significant decrease in ITZ-induced cytotoxicity with GSH pre-treatment. This protective effect was most pronounced at the 4-h time point; at 24 h, there was minimal difference between the 50 and 500 μM GSH groups. The reason for this is unknown, but it is possible that by the end of the experiment, the cells had reached their maximum capacity to uptake or respond to GSH, regardless of the amount provided. The protective effect of GSH was also seen in vehicle control cells, which likely experienced some oxidative stress attributable to the toxic effects of the DMSO vehicle (34). The toxic effect of DMSO is evident in Figure 3 in the 0 μM ITZ treatment groups, which contained no ITZ but were treated with the DMSO vehicle. In the neutral red assay, cytotoxicity is normalized to an untreated control that does not contain any additives, only culture media. Therefore, any amount of cytotoxicity observed in the 0 μM ITZ groups represent that induced by DMSO.

The benefit of GSH pre-treatment demonstrated here provides additional evidence that oxidative damage likely contributes to ITZ-associated hepatotoxicity. Studies in rodent models have demonstrated significant alterations in hepatic redox status during ITZ treatment including increased hepatic myeloperoxidase, nitric oxide, and malondialdehyde as well as decreased superoxide dismutase, glutathione peroxidase, and reduced glutathione concentrations (14, 15). These findings, combined with the protective effect of GSH pre-treatment documented in our study, support a potential role for GSH in the cellular response to ITZ-associated hepatotoxicity; however, the exact mechanism remains unknown.

One possible mechanism for GSH to reduce cytotoxicity is by binding and detoxifying toxic ITZ metabolites. A similar mechanism has been proposed for KTZ hepatotoxicity in which its metabolite is oxidized to reactive metabolites that bind hepatic proteins (35–37). GSH significantly reduces KTZ metabolite-protein complexes by binding the reactive sites on KTZ derivatives, promoting elimination (38). Toxic ITZ metabolites could be produced similarly but have yet to be identified. ITZ undergoes extensive hepatic metabolism yielding >30 metabolites in humans (31). The major metabolite in humans and rodents, hydroxy-ITZ (OH-ITZ), is produced by cytochrome P450 (CYP) 3A4. OH-ITZ is also the major metabolite in canine serum (39), suggesting that a similar but, as of yet, undefined pathway exists in dogs. In rodents, CYP induction decreases ITZ-associated hepatotoxicity while CYP inhibition increases toxicity, so either the parent compound or metabolites produced by an alternative pathway likely cause toxicity (32, 40). Interestingly, ITZ itself is a CYP inhibitor (40–43), so it is possible that autoinhibition of its own metabolizing CYP enzymes could potentiate toxicity over time.

This study sets the groundwork for additional investigation into the use of antioxidants in ITZ-associated hepatotoxicity in dogs. There are, however, some limitations to these *in vitro* experiments. A limitation inherent to all *in vitro* experiments is that our results may not reflect *in vivo* biologic behavior in dogs experiencing clinical ITZ-associated hepatotoxicity. To address this, we chose primary hepatocytes cultured in a sandwich configuration as this is the preferred method for *in vitro* drug disposition and hepatotoxicity studies (44). Hepatocytes were pre-incubated with GSH to achieve maximum effect despite pre-treatment not being a practical expectation in dogs requiring immediate antifungal therapy. However, daily co-administration of GSH precursors with ITZ *in vivo* is expected to provide a long-term effect over time. Future work will also incorporate functional parameters such as albumin production and ammonia tolerance as well as markers of cell redox status (reduced GSH, superoxide dismutase, catalase). Additionally, primary hepatocytes were obtained from a single donor, so future studies will investigate inter-individual variation in ITZ toxicity *in vitro* and *in vivo*. We also plan to investigate the effect of GSH on ITZ efficacy in fungal culture to determine if the antifungal effects of ITZ are altered in an antioxidant rich environment. Lastly, the potential beneficial effects GSH and other antioxidants in ITZ-associated hepatotoxicity should be confirmed in clinical trials.

Here, we showed that ITZ causes dose- and time-dependent hepatocyte toxicity *in vitro*. These findings demonstrate that primary canine hepatocytes are a viable model in which to study ITZ-associated hepatotoxicity in dogs. Neutral red uptake appears to be the most suitable measurement of cell viability in canine hepatocytes exposed to ITZ; this model can be used in future studies investigating the metabolism of ITZ and the effect of various antioxidants on toxicity. Most importantly, ITZ-induced cytotoxicity is mitigated by pre-incubation with GSH, suggesting a possible role for GSH or its precursors in the management of ITZ-associated hepatotoxicity in dogs. Results of this and future studies are critical to developing guidelines for the rational use of antioxidants in ITZ-associated hepatotoxicity in dogs.

## Data Availability Statement

The raw data supporting the conclusions of this article will be made available by the authors, without undue reservation.

## Ethics Statement

Ethical review and approval was not required for the animal study because this was an *in vitro* study using commercially purchased cells.

## Author's Note

Some data and figures presented in this manuscript were previously presented as an abstract at the American College of Veterinary Pathologists Annual Meeting 2019 in San Antonio, TX and in the Journal of the Federation of American Societies for Experimental Biology (45). The content of this manuscript has been published as part of the thesis of NK (46).

## Author Contributions

NK and JR contributed to study design, sample collection, data analysis, and manuscript preparation. MV and KS contributed to study design and approved the final manuscript. All authors contributed to and approved the final manuscript.

## Conflict of Interest

The authors declare that the research was conducted in the absence of any commercial or financial relationships that could be construed as a potential conflict of interest.

## Abbreviations

ALT: alanine aminotransferase
DMSO: dimethyl sulfoxide
GSH: glutathione
ITZ: itraconazole
SAMe: S-adenosyl-L-methionine.
